# Large-scale sequencing study of de novo regulatory Tandem Repeats (TRs) identifies new ASD (Autism Spectrum Disorders) candidate genes integrating gene expression mapping, brain scRNA-seq and organoid models

**DOI:** 10.21203/rs.3.rs-8374597/v1

**Published:** 2026-02-13

**Authors:** Maria Cristina Rodriguez Fontenla, Pablo Carballo-Pacoret, Sara Dominguez-Alonso, Javier Gonzalez-Peñas, Mara Parellada, Celso Arango, Angel Carracedo

**Affiliations:** University of Santiago de Compostela; Hospital General Universitario Gregorio Marañón, IiSGM, CIBERSAM, School of Medicine, UCM; Hospital General Universitario Gregorio Marañón, IiSGM, CIBERSAM, School of Medicine, UCM; Hospital General Universitario Gregorio Marañón, IiSGM, CIBERSAM, School of Medicine, UCM; Hospital General Universitario Gregorio Marañón, IiSGM, CIBERSAM, School of Medicine, UCM; Department of Chid Psychiatry. Hospital Universitario La Paz. IdiPAZ. School of Medicine. Universidad Autónoma de Madrid. CIBERSAM; Universidad de Santiago de Compostela (CIMUS) and SERGAS

## Abstract

In this study, we performed an integrative analysis of de novo tandem repeats (TRs) to unravel the missing heritability that may be hidden in 85,394 active cis-regulatory elements (cCREs) from ENCODE through target sequencing in a Spanish cohort of 200 ASD trios, using a robust bioinformatic pipeline. For the integrative analysis, we use data from 1,637 ASD simplex quad families from the Simons Simplex Collection (SSC). We then incorporated multiple layers of functional annotation, including predicted transcription factor (TF) binding sites, gene mapping based on physical proximity and expression correlation, pathogenicity scoring, single-cell RNA-seq data from human brain in ASD cases and controls and cortical organoid expression data.

Together, our analyses identified multiple ASD-relevant candidate genes supported by convergent lines of evidence. Notably, *ECHS1* emerged as a strong candidate, affected by several de novo TRs in both the Spanish cohort and the SSC. It was also identified as the most significantly associated gene through expression-based gene mapping (T-Gene) and showed consistent differential expression in excitatory neurons of the cerebral cortex at the single-cell level along with increased expression in late-stage cortical organoids.

These findings remark the value of integrating genetic and transcriptomic information to improve the identification of potential risk genes for ASD, particularly within non-coding regions. Our approach also highlights the importance of identifying complex genetic variation, such as de novo TRs, that are typically missed in conventional exome or whole-genome analyses, and require specialized bioinformatic strategies for accurate detection and interpretation.

## INTRODUCTION

Autism Spectrum Disorders (ASD) are neurodevelopmental disorders (NDDs), characterised by repetitive or restrictive behaviours, atypical social function and communication deficits (1,2). ASD have a high genetic component with an estimated heritability of around 80% (3). Although several autism-associated genes and variants have been identified through various genetic approaches—including GWAS (4), WES (5), WGS (6), and integrative omic analyses such as single-cell RNA sequencing (scRNA-seq) (7), these studies still fall short of accounting for the full genetic contribution estimated in family and twin studies. Thus, a substantial fraction of the so-called “missing heritability” remains unexplained. Several factors may contribute to missing heritability in ASD, including sample heterogeneity, undetected ultra rare variants, variants lying within regulatory regions, somatic and postzygotic mutations and complex structural variation such as tandem repeats and mobile elements. Detecting many of these variants often requires specialized sequencing technologies and bioinformatic tools.

Thus, de novo and inherited single-base mutations, small insertions and deletions (indels) or large copy number variations (CNVs) can be identified under conventional sequencing and routine genetic diagnosis screening procedures. However, part of this missing heritability may stem from the fact that current bioinformatics pipelines often discard information from repetitive regions which are challenging to interpret using short-read sequencing methods (8,9).

Tandem repeats (TRs) are sequences of bp motifs that are repeated consecutively a specific number of times in the DNA sequence. They vary both in the sequence of the repeated motif and in the number of repeat units. Motif repeats shorter than 100 bp represent approximately 3% of the human genome (10), with over a million known TRs distributed throughout the genome.

The classification of TRs has always been diverse, as they are usually classified according to their length (expansion or contraction of the repeat motif), location and/or frequency of occurrence: microsatellites (STRs) with repeat motifs shorter than 5 bp, are usually present throughout the genome. Minisatellites, with repeat motifs ranging from 5 bp to 100 bp, are less common. Satellite DNA, found at centromeres, consists of 6 bp motifs repeated between 300 and 8,000 times, while tandem repeats also consisting of 6 bp motifs, are repeated between 2,000 and 50,000 times at the ends of chromosomes. In addition, motifs longer than 5bp, often called Variant Number Tandem Repeats (VNTRs), have also been studied for their role in human traits (11).

As we can see, the classification of TRs is complex. They are found in many parts of the genome, although they are predominantly located within non-coding regions. TRs accelerate evolution and adaptation due to their mutation rates, which are between 10 and 100,000 times higher than those of the rest of the genome (12). These high mutation rates create new copy lengths and copy numbers, contributing to genomic diversity and potentially giving rise to novel traits. In addition, TRs are involved in cellular processes such as cell cycle stability, telomere maintenance, or the regulation of gene expression. They are known to contribute to disease etiology by altering TF binding and three-dimensional chromatin structure, affecting differential gene expression (13).

For example, well-known repeats of TRs lead to diseases, such as the CAG repeat motif, which causes Huntington’s Disease (14), as well as other genetic disorders (15). In addition to autism, where the expansion of the CGG motif in the *FMR1* gene is a direct cause of Fragile X syndrome, a condition closely related to ASD (16), TRs are linked to several other diseases of the nervous system, such as ataxia, schizophrenia, amyotrophic lateral sclerosis (ALS) or dementia (17–20).

It is known that the number of copies of TRs (21) and structural variations are relevant in the aetiology of ASD (22,23), as it is also known that there is an increased variation in TRs in regulatory regions (11). There are studies that have investigated genome-wide tandem repeats (24,25), and others who have studied mutations in regulatory regions (26), underscoring the need for future research on the identification of regulatory target genes and neuronal populations involved using single-cell resolution, which has not yet been addressed.

In this study, we set out to investigate the role of regulatory de novo tandem repeats (TRs) in ASD risk by integrating genomic, epigenomic, and transcriptomic data. Specifically, our objectives were: (1) to identify de novo TR mutations within active cis-regulatory elements (cCREs) in a Spanish ASD cohort of 200 ASD trios. and validate their contribution using large-scale data from the SSC (1,637 ASD simplex quad families); and (2) to functionally prioritize these variants through multi-layered annotation, including predicted disruption of transcription factor binding, expression-based gene mapping, pathogenicity scoring, biological pathways, single-cell transcriptomic signatures from the ASD human brain vs controls and expression patterns of target genes in cortical brain organoids.

This approach aims to uncover novel ASD risk genes within the regulatory genome and to highlight the contribution of complex regulatory variation that is often missed by conventional sequencing analyses.

## MATERIAL AND METHODS

### Spanish Cohort

The analysis presented in this study was conducted on a subset of 200 trios from the Spanish ASD cohort described in Alonso-Gonzalez et al. 2021 (27), which comprises 360 trios, each consisting of unaffected parents and an affected proband, from Fundación Pública de Medicina Xenómica, Santiago de Compostela and Hospital Universitario Gregorio Marañon, Madrid, Spain. Patients involved in the study were diagnosed by psychiatrists or neurologists, following the criteria of both the Diagnostic and Statistical Manual of Mental Disorders, Fourth Edition Text Revision (DSM-IV-TR) and Fifth Edition (DSM-5). When deemed necessary, the Autism Diagnostic Observation Schedule (ADOS) and the Autism Diagnostic Interview-Revised (ADI-R) were also administered. Patients with syndromic autism were excluded. All participants (probands, parents or legal representatives) gave their written consent and the study was conducted under the Declaration of Helsinki. The Galician Committee of Research Ethics (Xunta de Galicia) has approved this study under registration number 2020/400.

From the 360 trios forming the Spanish cohort with exome data, Copy Number Variation (CNV) array and phenotypic information, 200 trios were selected among those with negative results in the CNV array, following the procedure outlined in Alonso-Gonzalez et al., 2021 (27).

#### Selection of regulatory regions

Targeted sequencing (see next section) has been carried out on cis-regulatory regions (cCREs) from the ENCODE project (28) (version 2, https://screenv2.wenglab.org/), corresponding to approximately 1% of the total human genome, classified in 5 different categories: promoter-like signatures (PLS), proximal enhancer-like signatures (pELS), distal enhancer-like signatures (dELS), high DNase and H3K4me3 signals only (DNase–H3K4me3) and high DNase and CTCF signals only (CTCF-only). Brain and GI tissues with DNAse-Seq data available were selected, both from adult (n = 14) and embryonic tissue (n = 59). For each tissue, cCREs labeled as “Low-Dnase” were excluded, as they are inactive in the given tissue. From the total of 926,535 human cCREs, we selected those that exhibit activity in a greater number of the interrogated tissues. Thus, we selected cCREs that were active in 36 or more tissues (n = 85,394 cCREs).

#### Sequencing of regulatory regions

Targeted sequencing was done at the National Center for Genomic Analysis (CNAG) using the KAPA HyperChoice Target Enrichment custom probes and with an average coverage of 30·. Samples with sex discrepancies when compared to reported pedigrees were dropped and replaced, along with all other samples from the same family. Moreover, samples which failed CNAG’s quality control (DNA < 100 ng / critical degradation (genomic quality number (GQN) < 3.3)) were also removed and consequently substituted, leaving 71 trios from Santiago and 129 from Madrid. Sequencing reads were aligned to GRCh38/hg38 using the Burrows-Wheeler Aligner. Raw CRAM and vcf sequencing files have been transferred, stored and handled at the CESGA, Centro de Supercomputación de Galicia (https://www.cesga.es). Sequencing data are available at: EGA repository number: EGAS50000001395 https://ega-archive.org/studies/EGAS50000001395

#### Simons Simplex Collection cohort

To carry out a gene-level meta-analysis and a single-cell mapping analysis, we requested the STR dataset from the Simons Simplex Collection via the Simons Foundation Autism Research Initiative (SFARI) (https://base.sfari.org). We retrieved a CSV file containing detailed information on 175,291 mutations identified in 1,593 quad families (also used in Mitra et al.(29)). Genomic positions for STRs were overlapped with the cCREs analysed in our study using bedtools (https://github.com/arq5x/bedtools2), creating a new custom file only with cCREs that we had sequenced that had mutations in SSC cohort.

The subsequent gene annotation was done with this file as input. It should be noted that the TRs workflow applied in our cohort and in Mitra et al. is largely identical ensuring a high degree of methodological homogeneity.

#### Tandem Repeat (TR) Mutation Detection Workflow

**Figure 1:**
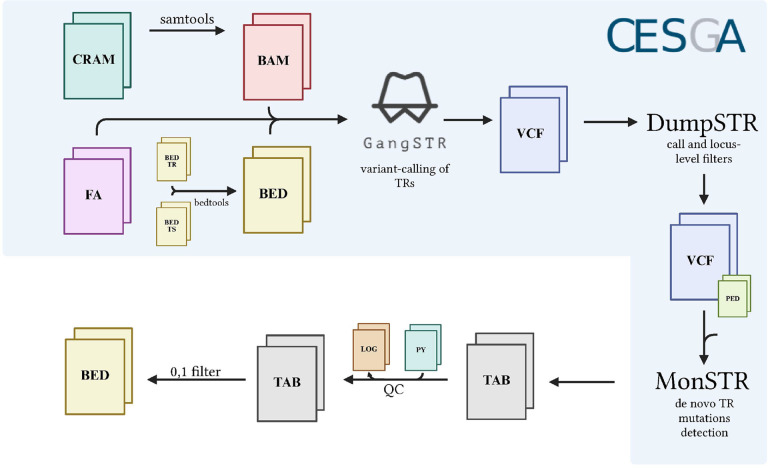
TRs mutations detection in Spanish cohort workflow. Bam, bed (custom TR catalogue) and fasta files were used as input for variant calling with GangSTR. The vcf output was used as input for call and locus-level filters with DumpSTR, and the vcf output was used as input for de novo TR mutations detection. Then, a quality control and filters were applied, resulting in a filtered bed file with TRs mutations.

The workflow (including file types, tools used, input and output formats, applied filters, and quality control steps performed in the analysis of the Spanish cohort), is summarized in Fig. 1. Custom TR catalogue elaboration and file transformation, calling of TRs, call and locus-level filters of variants and *in-silico* detection of TRs de novo variants, are found in *Supplementary Information*. A bed file with 107 mutations remained (*Supplementary Table 1*).

### Prioritizing pathogenic TR mutations

*SISTR (Selection Inference at Short TRs)* method described in Mitra et al., considers mutation, genetic drift and negative natural selection to calculate selection coefficients for each TRs. The parameter ‘*s*’ can be interpreted as the reduction in reproductive fitness for each repeat unit copy number, relative to the TR model allele in the population.

To estimate selection coefficients in the overlapping cCREs- TRs with the *GangSTR* catalogue, SISTR was run on the parental cohort (400 individuals).

Pathogenicity coefficients, which are allele-specific selection coefficients, have been calculated as follows: *(|a - opt|)s*, where *a* is the number of repeats for the de novo allele, *opt* is the optimal (or modal) number of repeats for that TR, and *s* is the selection coefficient for the TR calculated by SISTR in our parental cohort.

The input for SISTR was carried out using the statSTR tool from TR Tools package (https://github.com/gymrek-lab/TRTools). A single GangSTR vcf file was created, and statSTR was run with the filters --acount and --numcalled, to extract allele frequencies and total alleles. The resulting output was then merged with the GangSTR TR catalogue to obtain the input for running SISTR on the parental cohort.

#### Functional Annotation of TF Binding Sites

To determine whether de novo TR mutations disrupt transcription factor binding sites (TFBS) in the Spanish cohort, we have run the *FIMO* tool (30) (also from MEME Suite), designed to scan nucleotide sequences, to search for TF binding motifs. We selected the binding motifs of vertebrates from the JASPAR database (https://jaspar.elixir.no/downloads/) (31), that provides a catalog of TF motifs, obtained from validated experiments such as ChIP-Seq.

Two fasta files included sequences centered on the TR loci within or near SFARI genes, with one file representing the reference genome and the other incorporating the corresponding d*e novo* TR mutation sequences were generated. To this end, we have extended the mutated regions by ±15 bp to improve possible TF binding to overlapping regions between the flanking region and the mutation itself.

#### Integrative genetic analysis of de novo TRs from SSC collection and Spanish cohort

We aim to conduct a joint meta-analysis of microsatellite datasets from the Simons Simplex Collection (SSC) (25), (downloaded from base.sfari.org) and our Spanish cohort to identify overlapping genomic regions associated with ASD. The methods used in that study ensure homogeneity in the detection and annotation of TRs across both cohorts, allowing for reliable integration with our dataset.

Integrating these datasets increase statistical power, allow us to assess the consistency of observed signals across cohorts, and strengthen the robustness of candidate loci with potential functional relevance. Thus, a total of 200 trios of Spanish cohort + 1,637 quads from SSC and 107 de novo TRs mutations + 1305 from SSC were analysed. Overlapping cCREs between both cohorts were identified using *bedtools intersect*.

### Gene Mapping of Regulatory TRs using physical distance and expression data

To identify target genes based on genomic physical distance, we used the -closest function from the bedtools suite (https://bedtools.readthedocs.io/en/latest/content/tools/closest.html).

To identify target genes for TRs within cCREs, we used the T-Gene tool (32), which infers regulatory relationships by combining genomic closeness and ENCODE ChIP-seq expression data (hg19).

As input for both T-Gene and bedtools, we used the 57 cCREs carrying the 107 high-confidence de novo regulatory TRs from the Spanish cohort (*Supplementary Table 2*). The same was done with the SSC dataset (565 cCREs carrying 1305 mutations, *Supplementary Table 3*). A GTF annotation file (https://www.gencodegenes.org/human/release_7.html) corresponding to the hg19 genome assembly, was used as input in bedtools to ensure consistency between both analysis physical and expression.

To match this reference genome, TR coordinates from both the Spanish cohort and the SSC dataset from Mitra et al. were converted to hg19 using UCSC Table Browser (https://genome.ucsc.edu/cgibin/hgTables).

We customized T-Gene output to include all potential regulatory links by selecting the “Link” option, which avoids discarding cCREs that may share target genes. The output of T-gene containing regulatory genes for both cohorts is in *Supplementary Table 4* (Spanish cohort) and *5* (SSC cohort). For the Spanish cohort, genes were ordered to each analysed cCRE (*Supplementary Table 6*).

Subsequently, gene lists were filtered considering Correlation and Distance p-value (CnD p-value) < 0.05 of T-Gene. These filtered gene expression lists were used for distance vs expression analysis, GO enrichment and single-cell analysis.

For distance vs expression gene comparison, we filtered T-gene output taking in account only the most linked gene (lowest CnD p-value) for each cCRE for both cohorts.

In the Spanish cohort, we looked for genes related with SFARI database, to retain genes highly linked with ASD.

### GO enrichment analysis of TR associated genes

Gene Ontology enrichment analysis has been carried out on two platforms: Enrichr (https://maayanlab.cloud/Enrichr/) and ReVIGO (http://revigo.irb.hr). Enrichr is an online tool for enrichment analysis of gene sets; it was used to identify biological processes associated with genes related to our regulatory TRs by expression (T-Gene).

To carry out the GO enrichment analysis in the Spanish cohort, the output from T-Gene (*Supplementary Table 4*) was filtered to retain only those links with a CnD p-value < 0.05. The same was done for SSC cohort.

To carry out the GO enrichment analysis in both cohorts, we retain genes from T-gene output (*Supplementary Tables 4 and 5*) with CnD p-value < 0.5 without duplicated genes shared between cohorts.

For both cohorts and the metaanalysis with data from Mitra et al., we applied two thresholds: q-value < 0.1 and Bonferroni correction for multiple tests. Moreover, we have selected the top 30 Enrichr-enriched GO biological processes based on their p-value. Visualization was further refined using a custom R script adapted from ReVIGO.

### Single-Cell Gene Differential Gene Expression Analysis

In order to carry out a single cell differential gene expression analysis in neuronal and glial cells, we used ASD scGENE Portal (http://solo.bmap.ucla.edu/asdscgene/), resource that uses RNA-seq cell data from brain (33 ASD individuals and 31 control subjects) (33), comprising nearly 600,000 cell nuclei. We used this resource to explore whether any of the significant associated genes from both cohorts were expressed in specific neuronal or glial subtypes, and whether their expression was altered in ASD compared to controls.

There were 35 cell types identified by single-nucleus RNA sequencing (snRNA-seq), divided in 8 major cell types: oligodendrocyte progenitor cells (OPCs); astrocyte (ASTRO); microglia (MG); endothelial cells (ENDO); inhibitory neurons (INT); excitatory neurons (EXT); oligodendrocyte (ODC) and blood-brain barrier (BBB).

To carry out this analysis in the Spanish cohort, we retain genes from the T-gene output (*Supplementary Table 4*) with a CnD p-value < 0.05, and we looked for genes showing significant differential expression between ASD cases and controls defined by an FDR < 0.05, and an absolute log fold change (|logFC|) > 0.3.

For the dataset from Mitra et al., due to the larger number of candidate genes, we applied a slightly more stringent initial filter, selecting genes from the T-gene output (*Supplementary Table 5*) with a CnD p-value < 0.05 and a q-value < 0.1. These genes were then queried in the single-cell portal using the same differential expression thresholds as described above.

#### Gene expression trajectories across the differentiation of human cortical organoids in vitro and in vivo human brain development.

To explore the developmental trajectories of candidate gene expression in BrainSpan and brain cortical organoids, we used the Gene Expression tool (GECO, http://solo.bmap.ucla.edu/shiny/GECO/#), developed based on Gordon et al. (34).

### Visualization and Statistical Analysis Tool

RStudio v2024.12.0 + 467 was used to create the graphs presented, and the *circlize* library for RStudio was used for customized circo plot. Microsoft Excel 16.97 was used for the directionality bias graph.

## RESULTS

### SPANISH COHORT

#### Identification, Distribution, and Patterns of Regulatory de novo Tandem Repeats (TRs)

MonSTR identified 1,067 de novo non-strict de novo TRs mutations in the 200 families analysed from the Spanish cohort (*Supplementary Table 7*). After QC, 3 children and 6 TR loci were discarded and a list of 821 non-strict de novo TR mutations without outliers was obtained. After MonSTR prioritization, a total of 107 strict de novo TR mutations remained in 57 different cCREs, 83 different probands, out of the 200 analysed (after using GangSTR and MonSTR pipeline. See Methods section and *Supplementary Table 1*). The mean number of mutations per proband was 0.51, which is consistent with the genome-wide average of 53.9 mutations per individual reported by Mitra et al., taking in account that our analysis covers approximately 1% of the genome.

**Figure 2:**
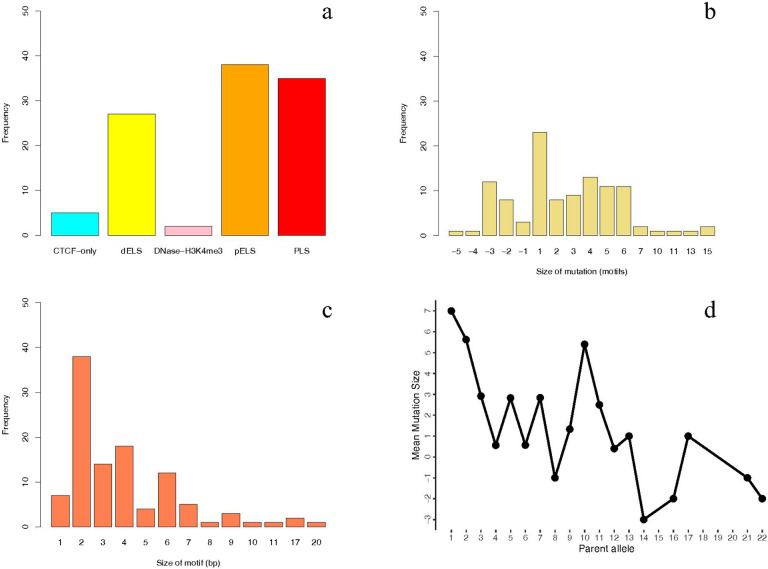
a)Bar plot displaying the ENCODE cCRE (candidate cis-regulatory elements) categories in which TR mutations were located in the Spanish cohort.b) Bar plot showing the distribution of de novo TRs by size, expressed as the number of repeat units gained or lost in the Spanish cohort; c) Bar plot showing the distribution of TR mutations according to motif length (e.g., mono-, di-, tri-nucleotide repeats…);d) Scatter plot illustrating the parental allele bias, with a tendency for shorter alleles to expand and longer alleles to contract

Analysing candidate cis-regulatory elements (cCREs), we found an enrichment in one particular cCRE category: proximal enhancer-like signature (pELS), with a total of 38 mutations out of 107 mutations (Fig. 2a).

Regarding the patterns of expansion, the most frequent pattern in the strict de novo TRs, involved the motif being repeated just once more than in the parent sequence. In contrast, the most common contraction mutation pattern was the deletion of three consecutive copies of the motif (Fig. 2b).

When analyzing the motif size of TRs in our cohort, we found that 38 out of the 107 mutations occurred in TRs with 2 bp motifs, making this the most frequent motif size in our dataset (Fig. 2c).

In our study, a significant bias towards expansions over contractions in de novo TRs was found. Specifically, 81 out of 107 (76%) were expansions while 26/107 (24%) were contractions (binomial test, n = 107, k = 81; p = 9.37×10^− 8^) (*See Supplementary Table 1*).

In addition, there is a directionality bias in mutation size that is observed in other studies as well. When the parental allele is short, the mutation tends to expand, and when the parental allele is large, it tends to contract. (Fig. 2d).

#### Prioritizing pathogenic TR mutations

Due to SISTR’s current limitation to repeat motifs of 2–4 base pairs, not all regions could be analyzed. Of the total regions considered, 7,561 regions were analysed using SISTR. 134 regions (1.8% could not be analysed, indicating that SISTR may assume certain patterns in the TRs that are not fulfilled at these loci). As a result, selection coefficients (‘s’) were successfully estimated for 7,427 loci (*Supplementary Table 8*).

Of the 107 de novo TR mutations identified, SISTR was able to calculate pathogenicity scores for 66. Among these, 13 mutations in 16 probands were in the top quartile of pathogenicity (score > 0.002655), and one mutation was linked with a SFARI gene (see Gene Mapping in the next section), *CASZ1* (See Supplementary *Table 9*). Detailed information for all 66 mutations analyzed with SISTR is provided in *Supplementary Table 10*.

#### Gene Mapping of Regulatory TRs by physical distance and expression data

As we mentioned in Material and Methods section, 57 cCREs with TRs mutations in the Spanish cohort were associated with their putative target genes using expression data of T-Gene (*Supplementary Table 4*).

For the distance vs expression comparison, T-gene links with CnD p-value > 0.05 were discarded, so 53 cCREs finally remain, because 4 cCREs did not have any link with CnD p-value < 0.05 (see Material and Methods and *Supplementary Table 11*). Closest genes by physical distance using bedtools are listed in *Supplementary Table 12*.

These 53 cCREs were used to compare whether the regulated gene was the closest physically or not. In nearly half of the cCREs (21 out of 53; 39.6%), the gene most significantly associated with the regulatory region was not the closest gene in terms of physical distance (*See Supplementary Table 13*).

Moreover, in the Spanish cohort, 9 mutations affecting 8 different cCREs were linked to genes listed in the Simons Foundation Autism Research Initiative (SFARI) database (Banerjee-Basu & Packer, 2010), the most comprehensive and reliable resource for ASD-associated genes (see *Supplementary Table 14*).

#### Functional Annotation of TF (Transcription Factor) Binding Sites

The results of looking at the cCREs with a linked SFARI gene have shown that in most cases (80%), TFs predicted to have the strongest binding to the mutated regions are the same as those predicted to bind the unmutated regions. The majority of these top-binding TFs belong to the *ZNF (zinc finger)* family.

However, we identified two *de novo* TRs that result in a change in the TF with the highest predicted binding affinity. The first TR affects TF binding by replacing *KLF9* with *Nrf1*, a gene previously studied in the context of neurodevelopmental disorders which belongs to a family of TFs previously related to neuronal functions. In the second case, a de novo TR alters binding from *ZNF384* to *EWSR1-FLI1* (*Supplementary Table 15*).

#### GO enrichment analysis of TR associated genes

GO enrichment analysis was performed using the genes linked to cCREs with a CnD p-value < 0.05 identified in the T-Gene output for Spanish cohort (n = 122) (See *Supplementary Table 16*).

No GO term was significant (q-value < 0.1). However, when applying the Bonferroni correction for multiple testing (adjusted significance threshold = 0.05/121 ≈ 0.00041), two GO terms were significantly enriched: *Regulation of Cell Differentiation* (GO:0045595, p-value = 0.00024) and *Negative Regulation of Astrocyte Differentiation* (GO:0048712, p-value = 0.00036) (*Supplementary Table 17*).

The top 30 biological processes by p-value from GO enrichment analysis of Enrichr, using ReVIGO for visualizing Spanish cohort genes are shown in *Supplementary Fig. 1*.

#### Single-Cell Gene Differential Gene Expression Analysis

scRNA-seq analysis in the Spanish cohort was done with the 122 genes linked to cCRE with CnD p-value < 0.05, the same used for the GO analysis (*Supplementary Table 16*). Single cell gene differential gene expression analysis revealed differential expression of several genes (*Supplementary Table 18*). Among them, *ECHS1,CALY, KTN1* and *THRA* were overexpressed in cases, while *NR4A1, PHLDB2, UBSK, KLHL32, PBX1, GLI3, NRG1 and FARP1* were underexpressed in cases. *C9orf3* was underexpressed in cases in oligodendrocytes and overexpressed in excitatory neurons. *NRG1* shows the greatest degree of underexpression in cases from the Spanish cohort (*logFC=−*1,016; *FDR* = 5.69^e − 14^; *cell type = EXT_9_L6)*.

*GLI3* was underexpressed in astrocytes *(logFC=−0.397, FDR = 1.26 × 10*^*− 4*^*), THRA* was overexpressed in excitatory neurons *(logFC = 0.397, FDR = 1.97 × 10*^*− 3*^) and *PBX1* was underexpressed in several cell types, being astrocytes the most underexpressed cell type (logFC=−0.484, FDR = *5.33 × 10*^*− 5*^). These three genes met the thresholds for differential expression and are present in the SFARI database (*Supplementary Table 5*). Genes that did not met these thresholds were excluded in subsequent analysis.

#### Integration of results in the Spanish cohort

**Figure 3:**
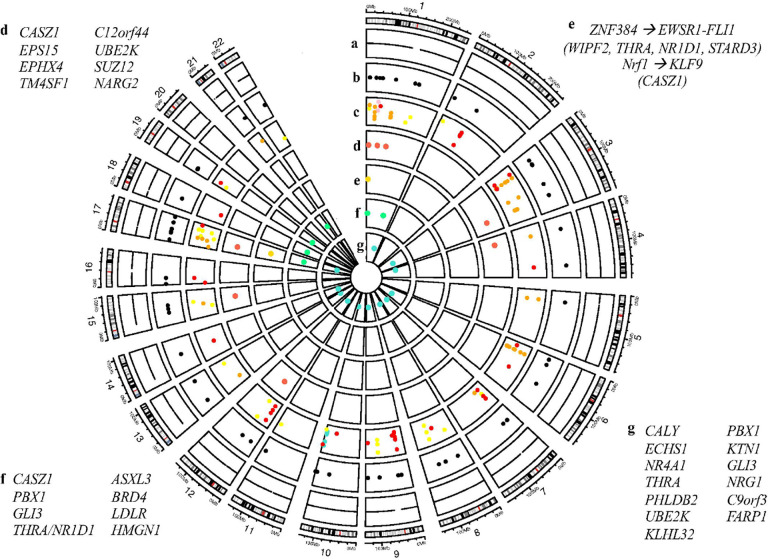
Integration of results from the Spanish cohort (genetic and functional annotations). From outside to inside: a) Sequenced cCREs; b) de novo TRs; c) ENCODE categories of cCREs; d) Genes with top pathogenicity scores; e) de novo TRs predicted to alter TF binding changes; f) SFARI genes; g) Genes differentially expressed between ASD cases and controls at scRNA-seq analysis.

This integrative circoplot (Fig. 3) highlights the convergence of de novo TRs mutations, regulatory genomic context (non coding regions), and gene expression signatures at the single-cell level. Notably, several high-priority genes (e.g., *ECHS1, CASZ1, PBX1)* appear in multiple layers of the analysis, suggesting that they are strong candidates supported at genetic and functional annotation levels. Furthermore, predicted TF binding alterations driven by TR mutations, offering a potential mechanistic link between non-coding variation and transcriptional dysregulation were also represented. The integration of single-cell transcriptomic data strengthens the relevance of these findings by providing cell-type-resolved evidence of dysregulation. It is important to remark that *ECHS1* is not only affected by TR mutations in regulatory regions, but also shows altered expression in ASD cases versus controls, reinforcing its candidacy as a neurodevelopmental risk gene.

### INTEGRATIVE ANALYSIS : SIMONS SIMPLEX COLLECTION AND THE SPANISH COHORT

#### Integrative genetic analysis of de novo TRs from SSC collection and Spanish cohort

Seven cCREs were overlapped between both cohorts considering that Mitra et al. 2021 carried out a WGS analysis (*Supplementary Table 19*).

#### Gene Mapping of Regulatory TRs by physical distance and expression data

As we mentioned in Material and Methods section, 565 cCREs with TRs mutations in the SSC cohort were associated with their putative target genes using expression data of T-Gene (*Supplementary Table 5*).

For the distance vs expression comparison, T-gene links with CnD p-value > 0.05 were discarded, so 487 cCREs finally remain, because 78 cCREs did not have any link with CnD p-value < 0.05 (see Material and Methods and *Supplementary Table 20*). Closest genes by physical distance using bedtools are listed in *Supplementary Table 21*.

These 487 cCREs were used to compare whether the regulated gene was the closest physically or not. Among the most significantly associated genes for these 487 cCREs, 162 (33.3%) were not the nearest gene based on physical distance (*Supplementary Table 22*).

If we remain with the most significant gene linked to each cCRE both of our cohort and from Mitra et al., 179/530 of the genes weren’t the closest to the cCRE (33.8%).

Ten regulatory genes were shared between the Spanish cohort and the SSC collection, taking in account the most significant genes linked with cCREs with CnD p-value < 0.05): *ECHS1, C9orf3, C9orf72, FARP1, KIAA0922, PBX1, SOCS3, UBE2K, THNSL2* and *WIPF2* (*Supplementary Table 23*).

#### GO enrichment analysis of TR associated genes

As we commented in the Spanish cohort, GO enrichment analysis was performed using the genes linked to cCREs with a CnD p-value < 0.05 identified in the T-Gene output of SSC cohort (n = 1125) (*Supplementary Table 24*).

In the SSC cohort, no GO term was significant (q-value < 0.1). When applying the Bonferroni correction for multiple testing (adjusted significance threshold = 0.05/3268 ≈ 0.00002), no GO term was significant either (*Supplementary Table 25*).

When the Spanish cohort was meta-analysed together with the SSC data, without duplicated genes shared between cohorts (n = 1220 genes) (*Supplementary Table 26*), the significant GO terms (q-value < 0.1) were: *Positive Regulation of Transcription by RNA Polymerase II* (GO:0045944, q-value = 0.0099); *Positive Regulation of DNA-templated Transcription* (GO:0045893, q-value = 0.3475) and *Neural Tube Closure* (GO: 0001843, q-value = 0.0567) (*Supplementary Table 27*).

If we apply Bonferroni correction (0.05/212 = 0.00024), the significant GO terms were the same as above, plus *Primary Neural Tube Formation* (GO:0014020, p-value = 0.00019) (*Supplementary Table 27*).

In this analysis, 27 genes were shared between cohorts (*Supplementary Table 28*), including *ECHS1*.

**Figure 4:**
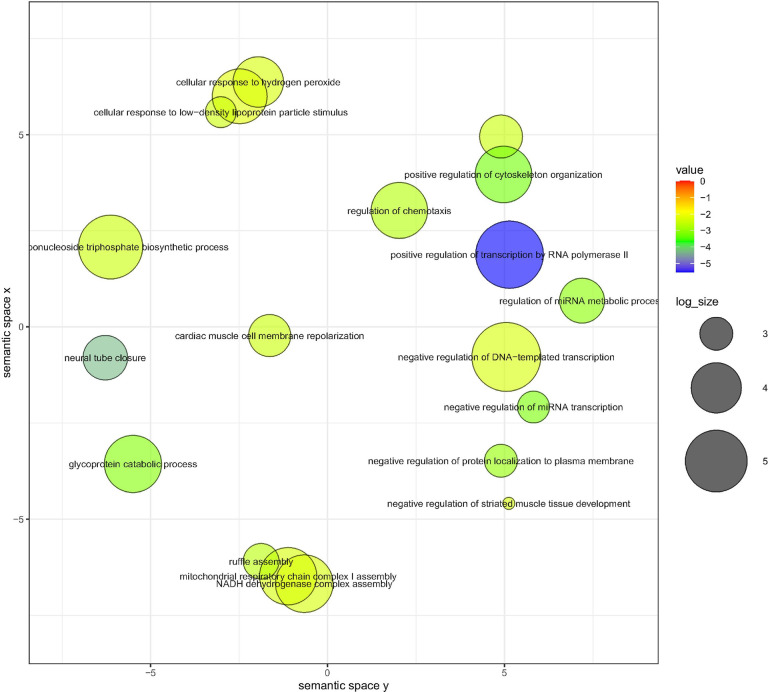
Dot plot showing the top 30 most significant biological processes related with both genes linked to Spanish TRs mutations and genes linked to TRs mutations from SSC cohort.

The top 30 biological processes by p-value from GO enrichment analysis of Enrichr, using ReVIGO for visualizing genes from both cohorts (integrative analysis) are shown in Fig. 4.

#### Single-Cell Gene Differential Gene Expression Analysis

The same analysis of scRNA-seq done in the Spanish cohort, was done with linked genes from the SSC cohort with CnD p-value < 0.05 and q-value < 0,1 (n = 202), as we discussed above (*Supplementary Table 28*). Results have shown that the most upregulated gene was *PTGES3* (*logFC = 0.855; FDR = 1.60×10*^*− 10*^; *cell type = EXT_9_L6*), while the most downregulated was *RERE (logFC= −0,647; FDR = 5.87×10*^*− 8*^; *cell type = EXT_9_L6* and l*ogFC=* −0,934; *FDR* = 3.93 *x10*^*− 6*^; *cell type = ODC_1)* (*Supplementary Table 29*).

When comparing gene mapping lists from the Spanish cohort (CnD p-value < 0.05, and the SSC cohort (CnD p-value > 0.05 and q-value < 0.1), 5 genes are shared between cohorts, *ECHS1, THNSL2, KIAA0922, PRRC2A* and *C9orf72* (*Supplementary Table 31*).

However single cell gene expression meta-analysis showed that only *ECHS1* was significantly differentially expressed in both our cohort and the SSC cohort, specifically in excitatory neurons from the cerebral cortex (*logFC = 0,343; FDR = 5.92×10*^*− 8*^; *cell type = EXT_4_L56*).

Despite being identified as pathogenic by SISTR and supported by expression data in both cohorts and SFARI classification, *CASZ1* did not exhibit significant differential expression between ASD cases and controls, nor cell-type-specific expression.

#### Integration of SSC and region in chromosome 10 emerges as the top candidate region

**Figure 6:**
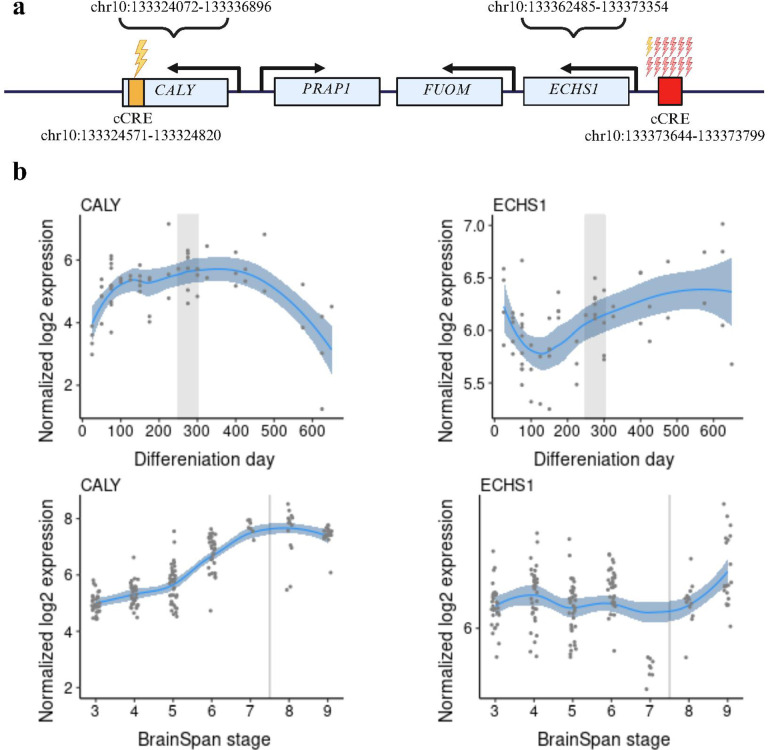
a) Region in chr10 with two mutated cCREs affecting regulation of CALY and ECHS1 genes (only mutated ccREs are shown). Yellow thunders represent Spanish cohort mutations, and red thunders SSC mutations. b) Developmental trajectories of ECHS1 and CALY expression in cortical organoids and BrainSpan. Transition from prenatal to post-natal stage with horizontal grey area/line.

*ECHS1 (enoyl-CoA hydratase, short chain 1)* is the only differentially expressed gene between ASD cases and controls when the targeted genes from de novo TRs in the Spanish and SSC cohorts are considered together in a single-cell analysis (Supplementary *Tables 18 and 29*). We found one individual with a mutation in the cCRE regulating *ECHS1* in the Spanish cohort and 11 individuals with 11 different mutations in the same cCRE in the SSC cohort (see *Supplementary Table 32*). Moreover, we discovered that this region is close to another mutated cCRE in the Spanish cohort linked to another gene, *CALY (Calcyon Neuron Specific Vesicular Protein)* (Fig. 6). We would like to point out that, as will be discussed in the next section, genes were assigned to mutated cCRES using T-Gene, an in silico method that does not employ ASD-specific expression data. Single-cell brain data from ASD cases and controls identified *ECHS1* as the unique gene, but *CALY*,located approximately 25000 bp upstream, was the most overexpressed when genes mutated in the Spanish cohort were considered (in interneurons and excitatory neurons) (see *Supplementary Table 2*). Together with the described function of *CALY*, the encoded protein interacts with the D1 dopamine receptor, this data suggests that the entire region would be an excellent candidate for future ASD functional studies.

We observed that *ECHS1* expression decreases during the early stages of differentiation of cortical organoids (~ day 100). After this point, expression progressively increases and remains elevated throughout later stages of neurodevelopment. This biphasic pattern suggests that *ECHS1* may have a role both in early neurodevelopmental transitions and in later maturation processes.

Across human brain development (Brain Span data) *ECHS1* shows relatively stable expression with a modest dip around stage 6 (late fetal/early postnatal), followed by an increase during later postnatal stages. In contrast, *CALY* expression markedly rose during early and mid-differentiation (100–300 days), and subsequently declined, suggesting predominant involvement in early neurodevelopmental processes. Together, these findings highlight distinct yet complementary temporal profiles: whereas *CALY* is most active during early neuronal specification and connectivity, *ECHS1* becomes increasingly relevant at later stages, underscoring how disruption of either process may contribute to ASD.

## Discussion

In our cohort, 57 different candidate cCREs and 107 high-confidence strict de novo TRs were identified. TRs demonstrated a large bias toward expansions (76%) over contractions, which is consistent with previous genome-wide studies. Interestingly, in contrast to the more prevalent single-repeat deletions previously documented, the most common contraction pattern in our dataset featured the deletion of three repeats (25). In addition, it was also found that there was a clear bias toward expansions among de novo TRs mutations. This pattern is consistent with prior genome-wide studies, which have also reported a higher prevalence of expansions over contractions among de novo TR mutations (25). From a functional perspective, this bias may have important biological implications. Expansions in regulatory regions could disrupt transcription factor binding motifs, alter chromatin accessibility, or interfere with the timing and levels of gene expression mechanisms during neurodevelopment (35). Importantly, the observed bias toward expansions was detected in a set of high-confidence, strictly de novo TRs, suggesting that the pattern is unlikely to result from technical artifacts. Instead, it likely reflects mutational patterns in the germline, potentially linked to chromatin architecture in regulatory regions. Moreover, it is important to note that this cohort consists exclusively of cases that have not been genetically diagnosed with autism through exome sequencing or CNV arrays but exhibit phenotypic traits consistent with ASD. This suggests that there must be some form of gene dysregulation that remains undetectable by the genetic diagnostic methods currently used. Supporting this, a recent genome-wide study identified over 2,000 VNTR loci enriched in regulatory regions, highlighting a potential source of genetic variation that escapes conventional detection (11).

In this Spanish cohort, a total of 107 de novo TRs mutations were identified. Using SISTR pathogenicity scores several genes were highlighted. *TM4SF1* (36) and *C12orf44* (37), which exhibit the highest pathogenicity scores, have been previously linked to neuronal defects but are not listed in the SFARI database. In addition, *CASZ1*, identified in a large-scale targeted sequencing study of neurodevelopmental disorders (38), is involved in neurodevelopment, regulating neurogenesis and the transition to gliogenesis (39), and is classified as a high-confidence ASD gene in SFARI. Other relevant genes also emerged, such as *TM4SF1* (40,41); *UBE2K* (42–44); and *NR4A1* (45,46).

The results from gene mapping analyses, based on physical proximity or expression data, underscore the importance of considering expression data when assigning genes to regulatory regions for further study. In nearly 40% of cases, the gene regulated by a cCRE was not the physically closest gene, indicating that the nearest gene is not always the one controlled by a regulatory region. These results remark the need to integrate three-dimensional genomic data (e.g., Hi-C, Capture-C) and expression-based approaches, rather than relying solely on linear distance, to assign regulatory elements to their target genes.

When the our cohort was analyzed independently, GO enrichment highlighted processes such as negative regulation of astrocyte differentiation and regulation of cell differentiation are especially relevant, underscoring the role of astrocytes in the etiology of ASD and pointing to the possible potential functional consequences of these mutations in early neural development and reinforcing the role of astrocytes in ASD etiology (47). Moreover, in the meta-analysis integrating both cohorts, several biological processes of early neurodevelopment were significantly overrepresented.

However, none of the genes classified as pathogenic by SISTR in the Spanish cohort were shared with the SSC collection. Using gene mapping with expression data to link cCREs carrying de novo TRs in both cohorts, we identified only ten genes with de novo TRs shared between both cohorts: *ECHS1, C9orf3, C9orf72, FARP1, KIAA0922, PBX1, SOCS3, UBE2K, THNSL2, and WIPF2*. Among these, only *ECHS1 (enoyl-CoA hydratase, short chain 1)* is differentially expressed at the single cell level, showing overexpression in excitatory neurons in ASD brain.

Taken together with the findings of Mitra et al., our results point to the involvement of novel genes that can only be uncovered through the application of TRs detection algorithms and subsequent analyses. Importantly, we also identify genes not represented in SFARI, suggesting the presence of complex structural repeat variants as well as rare, inherited variants in additional genes, further underscoring the genetic complexity underlying ASD. E*CHS1*, as we will discuss later, emerges as the main candidate gene because it is the only one shared between the Spanish cohort and the large SSC cohort at multiple analysis levels.

*ECHS1* is located near *CALY*, one of the target genes identified for de novo TRs in the Spanish cohort, which also showed overexpression in excitatory neurons in scRNAseq data. We found one individual carrying a mutation in the cCRE regulating *ECHS1* in the Spanish cohort, as well as 11 additional individuals with 11 distinct mutations in the SSC (29) which remarks the relevance of the region. It should be noted that genes were assigned to mutated cCRES using an *in silico* method that integrates generic ChIP-seq data from ENCODE and single-cell brain data from ASD cases vs controls, adding greater specificity to the ASD phenotype. We hypothesize that TRs within the cCREs analyzed in this study, or even in additional cCREs not yet included (as ENCODE v3 has identified a large number of novel cCREs in this region than the ENCODE v2 used in this study), may act as regulators of nearby genes independently *CALY* or *ECSH1*.

Among these two genes, *PRAP1 (proline-rich acidic protein 1)* and *FUOM (fucose mutarotase)* are also present; however, no mutations in our study targeted cCRES within these genes, and they are not expressed at the single-cell level in ASD brains nor controls.

Since the largest number of mutations are within *ECHS1*, we will thoroughly discuss its role without losing sight of the role of *CALY*, which encodes a dopamine receptor D1-interacting protein (48). The role of *CALY* in ASD or other NDDs has not been described or studied. However, dopamine receptors have been extensively investigated in ASD at the genetic and functional level. In particular, dopamine receptors D2/D3 in the striatum and D1 in the prefrontal cortex appear to be dysregulated in ASD (49). *ECHS1* encodes a mitochondrial enzyme with a key role in b-oxidation, as well as in the metabolic pathways of isoleucine and valine (50). Mutations in *ECHS1* are linked to a wide spectrum of clinical phenotypes, ranging from neonatal death to adult survival (51). The most common phenotype manifests like the Leigh syndrome (52) and related encephalopathies, characterised by different neurological impairments, arrhythmia and neonatal seizures (50). A second group of individuals manifest developmental regression resulting in severe developmental delay. Another group of related phenotypes include individuals with a normal development with paroxysmal dystonia that may be exacerbated by illness or exertion (53).In the context of ASD etiology, the overexpression of *ECHS1* observed in excitatory neurons may suggest that even subtle dysregulation of this mitochondrial pathway could contribute to altered neuronal activity. Given its essential role in energy metabolism, such changes may be particularly relevant to excitatory/inhibitory imbalance, a mechanism increasingly recognized in autism pathophysiology (54,55).

We aim to explore *ECHS1* and *CALY* together in cortical organoids at different times of differentiation to determine whether their regulation might be coordinated during neurodevelopment. Based on cortical organoid and Brain Span data, their timing of functional relevance differs: *CALY* appears to be relevant during early differentiation and synaptic formation while *ECHS1* seems to be more involved in later neuronal maturation and energy metabolism, consistent with its mitochondrial function. In terms of potential disease impact, dysregulation of *CALY* could disrupt early synaptic development and connectivity, impairing circuit formation, whereas dysregulation of *ECHS1* might compromise neuronal maturation processes, particularly through metabolic stress at later stages.

We hypothesize that if de novo TRs occur in regulatory regions in individuals with ASD, they may contribute to milder or intermediate phenotypes, in contrast to mutations in the coding region that are associated with severe syndromes as those described above. This suggests that noncoding TRs in regulatory regions of *ECHS1* might represent a mechanism underlying less severe, but clinically relevant, ASD-associated phenotypes.

The statistical strength of the SSC cohort further supports this gene as a promising target for genetic and functional studies aimed at clarifying how its dysregulation contributes to ASD pathophysiology.

### Limitations and Future Directions

While our study provides novel insights into the role of de novo TRs in regulatory regions and highlights *ECHS1* as a promising candidate gene, it should be noted that there are several limitations.

First, confirming the impact of TR mutations would require utilizing human-derived neuronal and glial models, such as brain organoids or iPSC-derived cells from the patients harboring the mutation. Second, although we leveraged large cohorts such as the Spanish cohort and SSC, there are important differences between them that may affect interpretation. For example, the Spanish cohort includes individuals with a prior specific exclusion/selection criteria, whereas the SSC cohort does not. These differences may slightly limit the comparability of our findings across populations, even when the methods used to identify variants are the same. Third, the assignment of regulatory elements to target genes relied on current annotations, which may not fully capture the complexity of three-dimensional genome architecture or long-range regulatory interactions.

It is also important to note that TRs larger than 150 bp are difficult to detect with established short-read sequencing platforms, as these are generally unable to accurately genotype large or complex repeat expansions (56). While specific short-read TR programs exist, they are currently limited in detecting repeats with very large motif sizes. Furthermore, genes were linked to cCREs containing mutations without accounting for TR characteristics, which may limit prioritization of certain regulatory elements over others.

Future studies should integrate multi-omics data, including epigenomic, proteomic, and functional assays, to better understand how TR-mediated dysregulation contributes to ASD. Targeted functional studies of *ECHS1* region will be important to explore how subtle changes in expression affect neuronal excitability and glial-neuronal interactions. Longitudinal analyses and studies in more diverse populations could further clarify how regulatory TRs contribute to the heterogeneity of ASD phenotypes, from mild to severe manifestations. Overall, addressing these limitations will provide a more comprehensive understanding of the genetic and cellular mechanisms underlying ASD and may identify novel therapeutic targets.

In conclusion, our study highlights the importance of de novo TRs in regulatory regions as contributors to ASD risk, uncovering candidate gene regions such as *ECHS1* that would have remained undetected using conventional approaches. By integrating genetic, transcriptomic, single-cell and cortical organoid data across cohorts, we provide new insights of these genes in neurodevelopment.

## Supplementary Material

Supplementary Files

This is a list of supplementary files associated with this preprint. Click to download.

- SupplementaryTables1.xlsx

## Figures and Tables

**Figure 5:**
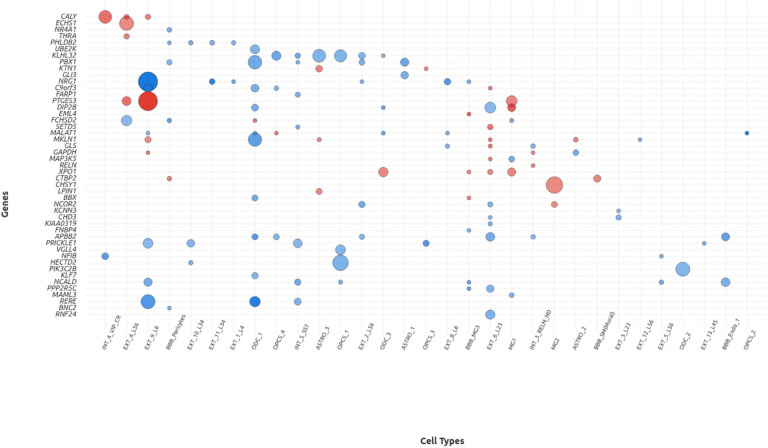
Single-cell differential expression dot plot displaying genes on the Y-axis analyzed in the Spanish cohort : CALY, ECHS1, NR4A1, THRA, PHLDB2, UBE2K, KLHL32, PBX1, KTN1, GLI3, NRG1, C9orf3, FARP1 (expression values provided in Supplementary Material), and in the SSC cohort (remaining genes on the Y-axis). NRG1 is the most downregulated gene in the Spanish cohort, while PTGSE3 is the most upregulated associated gene in the Simons Simplex Collection. Only ECHS1 shows significant association in both cohorts. Dot size corresponds to statistical significance (-log10 p-value), and color intensity reflects the magnitude of fold change between ASD and control samples, with red indicating upregulation in ASD and blue indicating downregulation.

## Data Availability

Sequencing data from the Spanish cohort are transferred to EGA (European Genome-phenome Archive) with Study Accession:EGAS50000001395.

## References

[1] American Psychiatric Association, American Psychiatric Association, editors. Diagnostic and statistical manual of mental disorders: DSM-5. 5th ed. Washington, D.C: American Psychiatric Association; 2013. 947 p.

[2] Rosti RO, Sadek AA, Vaux KK, Gleeson JG. The genetic landscape of autism spectrum disorders. Dev Med Child Neurol. 2014 Jan;56(1):12–8. 24116704 10.1111/dmcn.12278

[3] Sandin S, Lichtenstein P, Kuja-Halkola R, Hultman C, Larsson H, Reichenberg A. The Heritability of Autism Spectrum Disorder. JAMA. 2017 Sept 26;318(12):1182. 28973605 10.1001/jama.2017.12141 PMC5818813

[4] Autism Spectrum Disorder Working Group of the Psychiatric Genomics Consortium, BUPGEN, Major Depressive Disorder Working Group of the Psychiatric Genomics Consortium, 23andMe Research Team, Grove J, Ripke S, Identification of common genetic risk variants for autism spectrum disorder. Nat Genet. 2019 Mar;51(3):431–44. 30804558 10.1038/s41588-019-0344-8 PMC6454898

[5] Gaugler T, Klei L, Sanders SJ, Bodea CA, Goldberg AP, Lee AB, Most genetic risk for autism resides with common variation. Nat Genet. 2014 Aug;46(8):881–5. 25038753 10.1038/ng.3039 PMC4137411

[6] Sanders SJ, Murtha MT, Gupta AR, Murdoch JD, Raubeson MJ, Willsey AJ, De novo mutations revealed by whole-exome sequencing are strongly associated with autism. Nature. 2012 May;485(7397):237–41. 22495306 10.1038/nature10945 PMC3667984

[7] Quesnel-Vallières M, Weatheritt RJ, Cordes SP, Blencowe BJ. Autism spectrum disorder: insights into convergent mechanisms from transcriptomics. Nat Rev Genet. 2019 Jan;20(1):51–63. 30390048 10.1038/s41576-018-0066-2

[8] Bahlo M, Bennett MF, Degorski P, Tankard RM, Delatycki MB, Lockhart PJ. Recent advances in the detection of repeat expansions with short-read next-generation sequencing. F1000Research. 2018 June 13;7:736. 10.12688/f1000research.13980.1 PMC6008857 29946432

[9] Hannan AJ. Tandem repeat polymorphisms: modulators of disease susceptibility and candidates for ‘missing heritability’. Trends Genet. 2010 Feb;26(2):59–65. 20036436 10.1016/j.tig.2009.11.008

[10] International Human Genome Sequencing Consortium, Whitehead Institute for Biomedical Research, Center for Genome Research:, Lander ES, Linton LM, Birren B, Nusbaum C, Initial sequencing and analysis of the human genome. Nature. 2001 Feb 15;409(6822):860–921. 11237011 10.1038/35057062

[11] Zhang S, Song Q, Zhang P, Wang X, Guo R, Li Y, Genome-wide investigation of VNTR motif polymorphisms in 8,222 genomes: Implications for biological regulation and human traits. Cell Genomics. 2024 Dec;4(12):100699. 39609246 10.1016/j.xgen.2024.100699 PMC11701250

[12] Gymrek M, Willems T, Reich D, Erlich Y. Interpreting short tandem repeat variations in humans using mutational constraint. Nat Genet. 2017 Oct 1;49(10):1495–501. 28892063 10.1038/ng.3952 PMC5679271

[13] Liao X, Zhu W, Zhou J, Li H, Xu X, Zhang B, Repetitive DNA sequence detection and its role in the human genome. Commun Biol. 2023 Sept 19;6(1):954. 37726397 10.1038/s42003-023-05322-y PMC10509279

[14] Macdonald M. A novel gene containing a trinucleotide repeat that is expanded and unstable on Huntington’s disease chromosomes. Cell. 1993 Mar;72(6):971–83. 8458085 10.1016/0092-8674(93)90585-e

[15] Usdin K. The biological effects of simple tandem repeats: Lessons from the repeat expansion diseases: Table 1. Genome Res. 2008 July;18(7):1011–9. 18593815 10.1101/gr.070409.107 PMC3960014

[16] Fyke W, Velinov M. FMR1 and Autism, an Intriguing Connection Revisited. Genes. 2021 Aug 6;12(8):1218. 34440392 10.3390/genes12081218 PMC8394635

[17] Cortese A, Simone R, Sullivan R, Vandrovcova J, Tariq H, Yau WY, Biallelic expansion of an intronic repeat in RFC1 is a common cause of late-onset ataxia. Nat Genet. 2019 Apr;51(4):649–58. 30926972 10.1038/s41588-019-0372-4 PMC6709527

[18] Malik I, Kelley CP, Wang ET, Todd PK. Molecular mechanisms underlying nucleotide repeat expansion disorders. Nat Rev Mol Cell Biol. 2021 Sept;22(9):589–607. 34140671 10.1038/s41580-021-00382-6 PMC9612635

[19] Mojarad BA, Engchuan W, Trost B, Backstrom I, Yin Y, Thiruvahindrapuram B, Genome-wide tandem repeat expansions contribute to schizophrenia risk. Mol Psychiatry. 2022 Sept;27(9):3692–8. 35546631 10.1038/s41380-022-01575-x PMC9708556

[20] Trost B, Thiruvahindrapuram B, Chan AJS, Engchuan W, Higginbotham EJ, Howe JL, Genomic architecture of autism from comprehensive whole-genome sequence annotation. Cell. 2022 Nov;185(23):4409–4427.e18. 36368308 10.1016/j.cell.2022.10.009 PMC10726699

[21] Hannan AJ. Tandem repeats mediating genetic plasticity in health and disease. Nat Rev Genet. 2018 May;19(5):286–98. 29398703 10.1038/nrg.2017.115

[22] Brandler WM, Antaki D, Gujral M, Kleiber ML, Whitney J, Maile MS, Paternally inherited cisregulatory structural variants are associated with autism. Science. 2018 Apr 20;360(6386):327–31. 29674594 10.1126/science.aan2261 PMC6449150

[23] Marshall CR, Noor A, Vincent JB, Lionel AC, Feuk L, Skaug J, Structural Variation of Chromosomes in Autism Spectrum Disorder. Am J Hum Genet. 2008 Feb;82(2):477–88. 18252227 10.1016/j.ajhg.2007.12.009 PMC2426913

[24] Trost B, Engchuan W, Nguyen CM, Thiruvahindrapuram B, Dolzhenko E, Backstrom I, Genome-wide detection of tandem DNA repeats that are expanded in autism. Nature. 2020 Oct 1;586(7827):80–6. 32717741 10.1038/s41586-020-2579-z PMC9348607

[25] Mitra I, Huang B, Mousavi N, Ma N, Lamkin M, Yanicky R, Patterns of de novo tandem repeat mutations and their role in autism. Nature. 2021 Jan 14;589(7841):246–50. 33442040 10.1038/s41586-020-03078-7 PMC7810352

[26] Werling D, Brand H, An JY, Stone M, Glessner J, Zhu L, LIMITED CONTRIBUTION OF RARE, NONCODING VARIATION TO AUTISM SPECTRUM DISORDER FROM SEQUENCING OF 2,076 GENOMES IN QUARTET FAMILIES. Eur Neuropsychopharmacol. 2019;29:S784–5.

[27] Alonso-Gonzalez A, Calaza M, Amigo J, González-Peñas J, Martínez-Regueiro R, Fernández-Prieto M, Exploring the biological role of postzygotic and germinal de novo mutations in ASD. Sci Rep. 2021 Jan 11;11(1):319. 33431980 10.1038/s41598-020-79412-w PMC7801448

[28] The ENCODE Project Consortium, Abascal F, Acosta R, Addleman NJ, Adrian J, Afzal V, Expanded encyclopaedias of DNA elements in the human and mouse genomes. Nature. 2020 July 30;583(7818):699–710. 32728249 10.1038/s41586-020-2493-4 PMC7410828

[29] Mitra I, Huang B, Mousavi N, Ma N, Lamkin M, Yanicky R, Patterns of de novo tandem repeat mutations and their role in autism. Nature. 2021 Jan 14;589(7841):246–50. 33442040 10.1038/s41586-020-03078-7 PMC7810352

[30] Grant CE, Bailey TL, Noble WS. FIMO: scanning for occurrences of a given motif. Bioinformatics. 2011 Apr 1;27(7):1017–8. 21330290 10.1093/bioinformatics/btr064 PMC3065696

[31] Rauluseviciute I, Riudavets-Puig R, Blanc-Mathieu R, Castro-Mondragon JA, Ferenc K, Kumar V, JASPAR 2024: 20th anniversary of the open-access database of transcription factor binding profiles. Nucleic Acids Res. 2024 Jan 5;52(D1):D174–82. 37962376 10.1093/nar/gkad1059 PMC10767809

[32] O’Connor T, Grant CE, Bodén M, Bailey TL. T-Gene: improved target gene prediction. Wren J, editor. Bioinformatics. 2020 June 1;36(12):3902–4. 32246829 10.1093/bioinformatics/btaa227 PMC7320607

[33] Wamsley B, Bicks L, Cheng Y, Kawaguchi R, Quintero D, Margolis M, Molecular cascades and cell type–specific signatures in ASD revealed by single-cell genomics. Science. 2024 May 24;384(6698):eadh2602. 38781372 10.1126/science.adh2602

[34] Gordon A, Yoon SJ, Tran SS, Makinson CD, Park JY, Andersen J, Long-term maturation of human cortical organoids matches key early postnatal transitions. Nat Neurosci. 2021 Mar;24(3):331–42. 33619405 10.1038/s41593-021-00802-y PMC8109149

[35] Sun JX, Helgason A, Masson G, Ebenesersdóttir SS, Li H, Mallick S, A direct characterization of human mutation based on microsatellites. Nat Genet. 2012 Oct;44(10):1161–5. 22922873 10.1038/ng.2398 PMC3459271

[36] Shih SC, Zukauskas A, Li D, Liu G, Ang LH, Nagy JA, The L6 Protein TM4SF1 Is Critical for Endothelial Cell Function and Tumor Angiogenesis. Cancer Res. 2009 Apr 15;69(8):3272–7. 19351819 10.1158/0008-5472.CAN-08-4886 PMC2774109

[37] Guo T, Nan Z, Miao C, Jin X, Yang W, Wang Z, The autophagy-related gene Atg101 in Drosophila regulates both neuron and midgut homeostasis. J Biol Chem. 2019 Apr;294(14):5666–76. 30760524 10.1074/jbc.RA118.006069 PMC6462509

[38] Wang T, Hoekzema K, Vecchio D, Wu H, Sulovari A, Coe BP, Large-scale targeted sequencing identifies risk genes for neurodevelopmental disorders. Nat Commun. 2020 Oct 1;11(1):4932. 33004838 10.1038/s41467-020-18723-y PMC7530681

[39] Liu T, Li T, Ke S. Role of the CASZ1 transcription factor in tissue development and disease. Eur J Med Res. 2023 Dec 5;28(1):562. 38053207 10.1186/s40001-023-01548-y PMC10696751

[40] Kitajima H, Maruyama R, Niinuma T, Yamamoto E, Takasawa A, Takasawa K, TM4SF1-AS1 inhibits apoptosis by promoting stress granule formation in cancer cells. Cell Death Dis. 2023 July 13;14(7):424. 37443145 10.1038/s41419-023-05953-3 PMC10345132

[41] Tang Q, Chen J, Di Z, Yuan W, Zhou Z, Liu Z, TM4SF1 promotes EMT and cancer stemness via the Wnt/β-catenin/SOX2 pathway in colorectal cancer. J Exp Clin Cancer Res. 2020 Dec;39(1):232. 33153498 10.1186/s13046-020-01690-z PMC7643364

[42] Cai Y, Ji Y, Liu Y, Zhang D, Gong Z, Li L, Microglial circ-UBE2K exacerbates depression by regulating parental gene UBE2K via targeting HNRNPU. Theranostics. 2024;14(10):4058–75. 38994030 10.7150/thno.96890 PMC11234284

[43] Filatova EV, Shadrina MI, Alieva AKh, Kolacheva AA, Slominsky PA, Ugrumov MV. Expression analysis of genes of ubiquitin-proteasome protein degradation system in MPTP-induced mice models of early stages of Parkinson’s disease. Dokl Biochem Biophys. 2014 May;456(1):116–8. 24993970 10.1134/S1607672914030107

[44] Meiklejohn H, Mostaid MS, Luza S, Mancuso SG, Kang D, Atherton S, Blood and brain protein levels of ubiquitin-conjugating enzyme E2K (UBE2K) are elevated in individuals with schizophrenia. J Psychiatr Res. 2019 June;113:51–7. 30901725 10.1016/j.jpsychires.2019.03.005

[45] Li H, Zhao P, Xu Q, Shan S, Hu C, Qiu Z, The autism-related gene SNRPN regulates cortical and spine development via controlling nuclear receptor Nr4a1. Sci Rep. 2016 July 19;6(1):29878. 27430727 10.1038/srep29878 PMC4949425

[46] Okay K, Varış PÜ, Miral S, Ekinci B, Yaraş T, Karakülah G, Alternative splicing and gene co-expression network-based analysis of dizygotic twins with autism-spectrum disorder and their parents. Genomics. 2021 July;113(4):2561–71. 34087420 10.1016/j.ygeno.2021.05.038

[47] Allen M, Huang BS, Notaras MJ, Lodhi A, Barrio-Alonso E, Lituma PJ, Astrocytes derived from ASD individuals alter behavior and destabilize neuronal activity through aberrant Ca2 + signaling. Mol Psychiatry. 2022 May;27(5):2470–84. 35365802 10.1038/s41380-022-01486-x PMC9135629

[48] Ha CM, Park D, Han JK, Jang J ill, Park JY, Hwang EM, Calcyon Forms a Novel Ternary Complex with Dopamine D1 Receptor through PSD-95 Protein and Plays a Role in Dopamine Receptor Internalization. J Biol Chem. 2012 Sept;287(38):31813–22. 22843680 10.1074/jbc.M112.370601 PMC3442515

[49] Su P, Lai TKY, Lee FHF, Abela AR, Fletcher PJ, Liu F. Disruption of SynGAP–dopamine D1 receptor complexes alters actin and microtubule dynamics and impairs GABAergic interneuron migration. Sci Signal. 2019 Aug 6;12(593):eaau9122. 31387938 10.1126/scisignal.aau9122

[50] Masnada S, Parazzini C, Bini P, Barbarini M, Alberti L, Valente M, Phenotypic spectrum of short-chain enoyl-Coa hydratase-1 (ECHS1) deficiency. Eur J Paediatr Neurol. 2020 Sept;28:151–8. 32800686 10.1016/j.ejpn.2020.07.007

[51] Muntean C, Tripon F, Bogliș A, Bănescu C. Pathogenic Biallelic Mutations in ECHS1 in a Case with Short-Chain Enoyl-CoA Hydratase (SCEH) Deficiency-Case Report and Literature Review. Int J Environ Res Public Health. 2022 Feb 13;19(4):2088. 35206276 10.3390/ijerph19042088 PMC8871535

[52] Sakai C, Yamaguchi S, Sasaki M, Miyamoto Y, Matsushima Y, Goto Y ichi. ECHS1 Mutations Cause Combined Respiratory Chain Deficiency Resulting in Leigh Syndrome. Hum Mutat. 2015 Feb;36(2):232–9. 25393721 10.1002/humu.22730

[53] Olgiati S, Skorvanek M, Quadri M, Minneboo M, Graafland J, Breedveld GJ, Paroxysmal exercise-induced dystonia within the phenotypic spectrum of *ECHS1* deficiency: *ECHS1* Mutations, Dystonia, and PED. Mov Disord. 2016 July;31(7):1041–8. 27090768 10.1002/mds.26610

[54] Satterstrom FK, Kosmicki JA, Wang J, Breen MS, De Rubeis S, An JY, Large-Scale Exome Sequencing Study Implicates Both Developmental and Functional Changes in the Neurobiology of Autism. Cell. 2020 Feb;180(3):568–584.e23. 31981491 10.1016/j.cell.2019.12.036 PMC7250485

[55] Yizhar O, Fenno LE, Prigge M, Schneider F, Davidson TJ, O’Shea DJ, Neocortical excitation/inhibition balance in information processing and social dysfunction. Nature. 2011 Sept;477(7363):171–8. 21796121 10.1038/nature10360 PMC4155501

[56] Chintalaphani SR, Pineda SS, Deveson IW, Kumar KR. An update on the neurological short tandem repeat expansion disorders and the emergence of long-read sequencing diagnostics. Acta Neuropathol Commun. 2021 Dec;9(1):98. 34034831 10.1186/s40478-021-01201-x PMC8145836

